# Aliskiren Reduces Hepatic steatosis and Epididymal Fat Mass and Increases Skeletal Muscle Insulin Sensitivity in High-Fat Diet-Fed Mice

**DOI:** 10.1038/srep18899

**Published:** 2016-01-06

**Authors:** Kuei-Chuan Lee, Yun-Cheng Hsieh, Ying-Ying Yang, Che-Chang Chan, Yi-Hsiang Huang, Han-Chieh Lin

**Affiliations:** 1 Division of Gastroenterology and Hepatology, Department of Medicine, Taipei Veterans General Hospital, Taipei, Taiwan; 2 Department of Medicine, National Yang-Ming University School of Medicine, Taipei, Taiwan; 3 Institute of Clinical Medicine, National Yang-Ming University School of Medicine, Taipei, Taiwan; 4 Division of General Medicine, Department of Medicine, Taipei Veterans General Hospital, Taipei, Taiwan; 5 Division of Clinical Skill Training, Department of Medical Education, Taipei Veterans General Hospital.

## Abstract

Aliskiren has been found to reduce chronic injury and steatosis in the liver of methionine-choline-deficient (MCD) diet-fed mice. This study investigated whether aliskiren has an anti-steatotic effect in HFD-fed mice, which are more relevant to human patients with non-alcoholic fatty liver disease than MCD mice. Mice fed with 4-week normal chow or HFD randomly received aliskiren (50 mg/kg/day) or vehicle via osmotic minipumps for further 4 weeks. Aliskiren reduced systemic insulin resistance, hepatic steatosis, epididymal fat mass and increased gastrocnemius muscle glucose transporter type 4 levels with lower tissue angiotensin II levels in the HFD-fed mice. In addition, aliskiren lowered nuclear peroxisome proliferator-activated receptor gamma and its down-signaling molecules and increased cytochrome P450 4A14 and carnitine palmitoyltransferase 1A (CPT1a) in liver. In epididymal fat, aliskiren inhibited expressions of lipogenic genes, leading to decrease in fat mass, body weight, and serum levels of leptin and free fatty acid. Notably, in the gastrocnemius muscle, aliskiren increased phosphorylation of insulin receptor substrate 1 and Akt. Based on these beneficial effects on liver, peripheral fat and skeletal muscle, aliskiren is a promising therapeutic agent for patients with NAFLD.

Nonalcoholic fatty liver disease (NAFLD) is rapidly emerging as the most prevalent hepatic disorder in the Western world^1^. Some patients with NAFLD progress to non-alcoholic steatohepatitis, cirrhosis^2^, end-stage liver disease, and hepatocellular carcinoma^3^. There are currently no approved effective medications for the treatment of NAFLD, and the effectiveness of non-pharmacological treatments in this patient population is insufficient^4^. Thus, there is an urgent medical need for pharmacological agents for this disease.

Inhibition of the renin angiotensin system has been shown to improve NAFLD in animal studies^5^,^6^,^7^. However, the efficacy of angiotensin-converting enzyme inhibitors and angiotensin receptor blockers on human NAFLD is uncertain^8^. Genetic depletion of renin, the rate-determining enzyme for the generation of angiotensin, has been shown to result in resistance to diet-induced obesity and a reduction in fatty liver in mice^5^. Recently, aliskiren, the first renin inhibitor to be approved for clinical use, has been reported to reduce hepatic steatosis. Kishina *et al*.^9^ reported that aliskiren reduced hepatic steatosis in Shionogi-ob/ob mice, however the underlying mechanism to reduce steatosis was not elucidated and insulin resistance was not measured. We recently found that aliskiren attenuated hepatic steatosis by up-regulating fatty acid oxidation-related genes with increasing systemic insulin sensitivity in methionine-choline-deficient (MCD) diet-fed mice^10^. However, MCD mice have been reported to show better insulin sensitivity than normal mice^11^. This characteristic of MCD mice is in contrast to clinical patients with NAFLD, who often exhibit increased insulin resistance. Hence, it is still preliminary to translate the results from MCD mice or Shionogi-ob/ob mice to clinical use in patients with NAFLD.

Aliskiren has been demonstrated to improve insulin resistance in transgenic Ren2 rats, obese Zucker rats, streptozotocin-induced diabetic rats, and db/db mice^12^,^13^,^14^,^15^,^16^ by improving skeletal muscle glucose transport activity^13^,^15^,^16^ and pancreas islet function^12^. However, the effect of aliskiren on liver steatosis was not fully investigated in these studies. Therefore, this study aimed to evaluate the possible anti-steatotic effects and mechanisms of the chronic administration of aliskiren in a mouse model with hepatic steatosis and systemic insulin resistance which is relevant to clinical patients. Moreover, we also examined the epididymal fat and gastrocnemius muscle to investigate whether there was any effect of aliskiren on these two important tissues which contribute important roles to lipid metabolism.

## Results

### Aliskiren decreased lipid accumulation in liver

**Figure 1:**
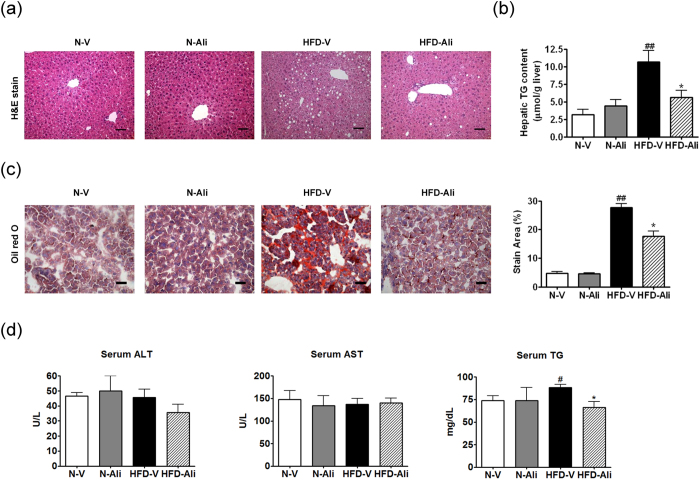
Aliskiren reduced hepatic steatosis in the mice fed with a high fat diet. (**a**) Hematoxylin and eosin stains (H&E stain) of liver, scale bar = 100 μm, (**b**) Hepatic triglyceride (TG) content, (**c**) Oil Red O stain and the quantification of hepatic steatosis, scale bar = 50 μm, (**d**) The serum levels of alanine aminotransferase (ALT), aspartate aminotransferase (AST) and triglyceride (TG) in all groups. N-V: mice fed with a normal diet (n = 6); N-Ali: mice fed with a normal diet and treated with aliskiren (n = 6); HFD-V: mice fed with a high fat diet; HFD-Ali (n = 7): mice fed with a high fat diet and treated with aliskiren (n = 7). ^#^p < 0.05 vs. the N-V group, ^##^p < 0.01 vs. the N-V group; *p < 0.05 vs. the HFD-V group.

The H&E and Oil Red O stains show that aliskiren decreased hepatic steatosis in the HFD-Ali group compared to the HFD-V group (Fig. 1a,c). The hepatic and serum triglyceride contents were significantly reduced after aliskiren treatment (Fig. 1b,d). There were no differences in the serum levels of alanine aminotransferase and aspartate aminotransferase among the groups (Fig. 1d).

### Aliskiren ameliorated systemic insulin resistance

**Figure 2:**
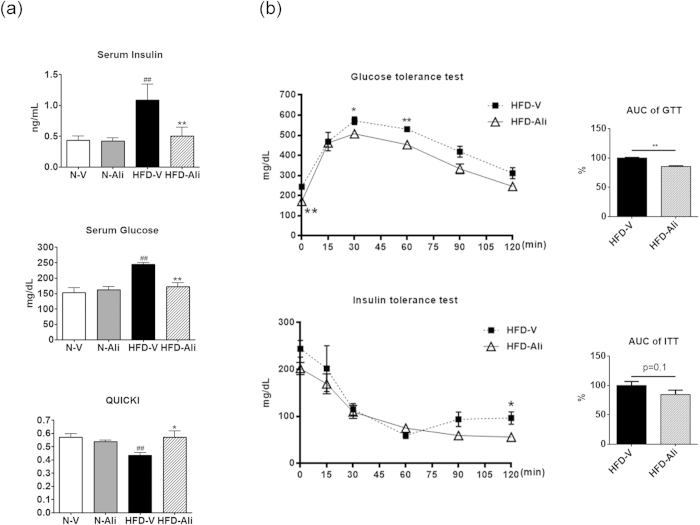
Aliskiren improved insulin sensitivity in the mice fed with a high fat diet. (**a**) The levels of insulin and glucose in serum and quantitative insulin sensitivity check index (QUICKI), a measurement of insulin sensitivity derived from the formula 1/(log insulin.glucose) from all groups. N-V: mice fed with a normal diet (n = 6); N-Ali: mice fed with a normal diet and treated with aliskiren (n = 6); HFD-V: mice fed with a high fat diet; HFD-Ali (n = 7): mice fed with a high fat diet and treated with aliskiren (n = 7). (**b**) Glucose tolerance test (GTT) and insulin tolerance test (IGG) in a set of HFD-V and HFD-Ali mice (n = 4 in each group). The area of the blood glucose response profile curve from each mouse is summed to obtain the area under the curve (AUC). The relative area values of the HFD-Ali group are expressed as a percentage relative to the average AUC of the HFD-V group, which is defined as 100%. ^##^P < 0.01 vs. N-V; *P < 0.05 vs. HFD-V; **P < 0.01 vs. HFD-V group.

The HFD-V mice exhibited higher serum levels of insulin and glucose and lower levels of quantitative insulin sensitivity check index (QUICKI) than the N-V group (Fig. 2a). Aliskiren treatment significantly decreased serum levels of insulin and glucose and increased QUICKI levels in the HFD-Ali group when compared to the HFD-V group. The HFD-Ali mice had a significant improvement in the glucose tolerance test and a trend of improvement in insulin tolerance test when compared with the HFD-V mice (Fig. 2b). It indicates that aliskiren treatment improves systemic insulin resistance in the HFD-fed mice.

### Aliskiren inactivated the PPARγ signaling pathway in the liver

**Figure 3:**
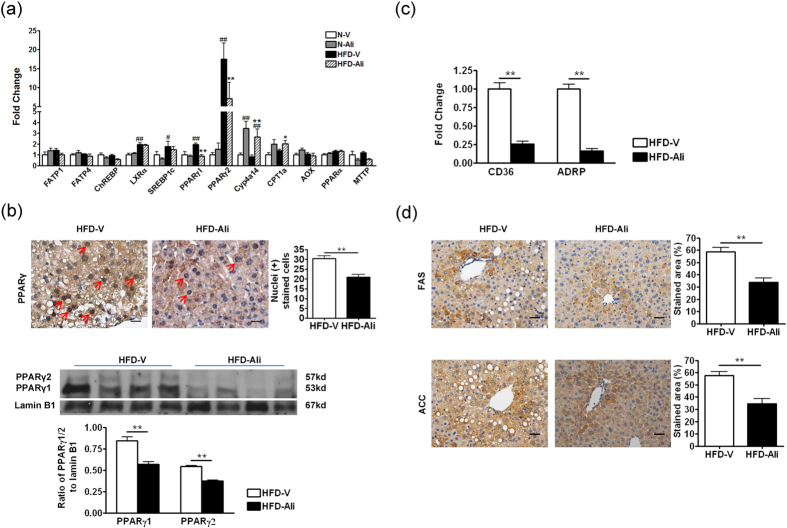
Aliskiren reduced hepatic steatosis through inactivating PPARγ and upregulating CYP4A14 and CPT1a. (**a**) RT-PCR of important genes involving in hepatic lipid metabolism. The results expressed were relative to the normal group, which was arbitrarily assigned a value of 1. Fatty acid transport protein 1/4 (FATP1/4), carbohydrate-responsive element-binding protein (ChREBP), liver X receptor alpha (LXRα), sterol regulatory element binding protein-1c (SREBP-1c), peroxisome proliferator-activated receptor alpha/gamma1/gamma2 (PPARα/γ1/γ2), cytochrome P450, family 4, subfamily a, polypeptide 14 (Cyp4a14), carnitine palmitoyltransferase 1a (CPT1a), acyl-CoA oxidase (AOX), microsomal triglyceride transfer protein (MTTP). N-V: mice fed with a normal diet (n = 6); N-Ali: mice fed with a normal diet and treated with aliskiren (n = 6). HFD-Ali (n = 7): mice fed with a high fat diet and treated with aliskiren (n = 7). (**b**) The immunohistochemical images of PPARγ and Western blot of nuclear PPARγ and lamin B1 in the HFD-V and HFD-Ali groups. Scale bar = 50 μm. Red arrows indicate positive nuclear stained cells. (**c**) The transcript expressions of fatty acid translocase (CD36) and adipose differentiation-related protein (ADRP) by RT-PCR in the HFD-V and HFD-Ali groups. The results expressed were relative to the HFD-V group, which was arbitrarily assigned a value of 1. (**d**) The immunohistochemical images of fatty acid synthase (FAS) and acetyl-CoA carboxylase (ACC) in HFD-V and HFD-Ali groups. Scale bar = 100 μm. ^#^P < 0.05 vs. N-V; ^##^P < 0.01 vs. N-V; *P < 0.05 vs. HFD-V; **P < 0.01 vs. HFD-V groups.

To elucidate which genes are involved in the anti-steatotic effect of aliskiren, the transcript expressions of the genes regulating hepatic lipid metabolism were measured by quantitative RT-PCR. Compared to the N-V mice, the HFD-V mice had significantly higher expressions of liver X receptor alpha (LXRα), sterol regulatory element binding protein-1c, and peroxisome proliferators activated receptor gamma 1/2 (PPARγ1/2) in the liver (Fig. 3a). Aliskiren treatment significantly decreased the hepatic expression levels of PPARγ1/2 (Fig. 3a). Immunohistochemically, it was demonstrated that aliskiren significantly reduced the nuclear staining of PPARγ in hepatocytes (Fig. 3b). Similarly, the nuclear protein expression of PPARγ1/2 was decreased significantly in the aliskiren-treated group (Fig. 3b). In addition, the down-signaling molecules of PPARγ such as free fatty acid uptake transporter (CD36), adipose differentiation-related protein (ADRP), fatty acid synthase (FAS) and acetyl-CoA carboxylase (ACC) were down-regulated by aliskiren (Fig. 3c,d). These findings suggest that aliskiren decreases hepatic lipogenesis by down-regulating a PPARγ signaling pathway.

On the other hand, aliskiren upregulated the transcript expression levels of CYP4A14 and CPT1a (Fig. 3a). These data suggest that on one hand, aliskiren may downregulate PPARγ to reduce de novo lipogenesis; on the other hand, it may stimulate CYP4A14 and CPT1a to promote fatty acid oxidation in liver.

### Aliskiren attenuated Ang II-associated oxidative stress and downregulated phosphorylation of Akt and AMPK

**Figure 4:**
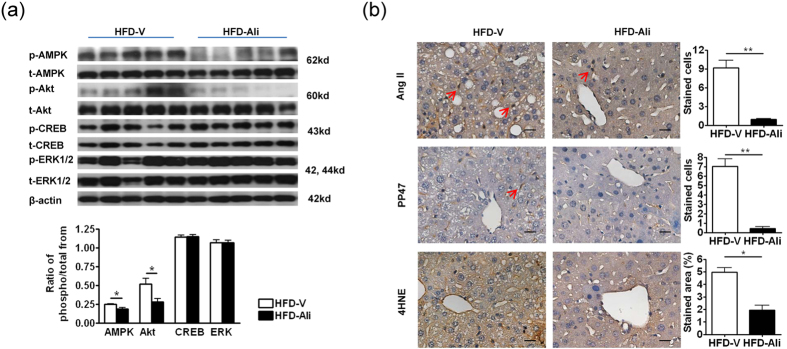
Aliskiren reduced phospho-Akt, phospho-AMPK and Ang II-induced oxidative stress. (**a**) Western blots of total or phosphorylated AMP-activated protein kinase (t/p-AMPK), protein kinase B (t/p-Akt), cAMP response element-binding protein (t/p-CREB), extracellular signal-regulated kinases (t/p-ERK) and beta-actin (β-actin) in the HFD-V and HFD-Ali groups. (**b**) The immunohistochemical images of angiotensin II (Ang II), phosphorylated p47 (pp47), and 4-hydroxynonenal (4HNE) in livers of the HFD-V and HFD-Ali groups. Scale bar = 50 μm, *p < 0.05 vs. the HFD-V group; **p < 0.01 vs. the HFD-V group.

The hepatic expressions of upstream mediators of PPARγ, the phosphorylation of Akt, AMPK, CREB and ERK1/2 were measured in the HFD-V and HFD-Ali mice. After aliskiren treatment, the phosphorylation of Akt and AMPK, but not the phosphorylation of CREB and ERK1/2 decreased significantly in the HFD-fed liver (Fig. 4a). It suggests that downregulation of p-Akt may contribute to the lower PPARγ expression in the aliskiren-treated liver.

To observe the renin-angiotensin inhibitory effect of aliskiren on liver, IHC stains of Ang II were examined and showed that aliskiren decreased Ang II expression in aliskiren-treated liver (Fig. 4b). Similarly, the expression levels of Ang II down-signaling phosphorylated p47 and 4-hydroxynonenal (4HNE) product were subsequently reduced after aliskiren treatment.

### Aliskiren reduced epididymal fat mass

**Figure 5:**
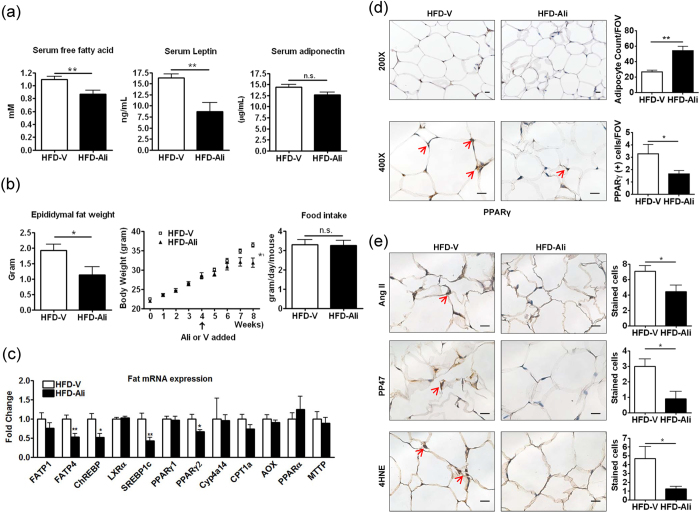
Aliskiren decreased weight, PPARgamma levels and Ang II-induced oxidative stress in epididymal fat. (**a**) The serum free fatty acid, adiponectin and leptin, (**b**) epididymal fat weight, body weight, and oral intake, (**c**) transcript expressions of genes regulating lipid metabolism in epididymal fat (abbreviations of genes: see Fig. 3 legend; the results expressed were relative to the HFD-V group, which was arbitrarily assigned a value of 1.), (**d**) immunohistochemical images of PPARγ and adipocyte number per field of view (FOV) in the epididymal fat tissue (scale bar = 50 μm), and **(e)** immunohistochemical images of angiotensin II (Ang II), phosphorylated p47 (pp47), and 4-hydroxynonenal (4HNE) in the HFD-V and HFD-Ali groups. Scale bar = 50 μm, *p < 0.05 vs. the HFD-V group; **p < 0.01 vs. the HFD-V group. n.s.: non-significant.

Because circulating free fatty acid also contributed to hepatic steatosis^17^, we investigated changes in its level. Aliskiren significantly decreased serum free fatty acid level in the HFD-Ali group (Fig. 5a). Peripheral fat is the most important source of serum free fatty acid^17^, and it was found that the HFD-Ali mice had significantly lower weights of epididymal fat tissue and the whole body without changes in the amount of oral intake (Fig. 5b). We then evaluated transcript expressions of genes regulating lipid metabolism in fat tissues. In contrast to the liver, the HFD-Ali epididymal fat tissues exhibited lower expression levels of FATP4, ChREBP, SREBP1c and PPARγ2 than the HFD-V group (Fig. 5c). The immunohistochemical images of the HFD-Ali fat tissues showed a reduction in the number of positive PPARγ-stained adipocytes and an increase in adipocyte counts which inversely correlated to the size of adipocytes (Fig. 5d). The expressions of angiotensin II, pp47, and 4HNE were decreased in the HFD-Ali epididymal fat tissues (Fig. 5e). Levels of the fat-secreted adipokines, leptin and adiponectin, were then evaluated. Aliskiren did not change the level of serum adiponectin but significantly reduced the level of leptin (Fig. 5a). These findings demonstrate that aliskiren inhibited expressions of lipogenic genes and Ang II in epididymal fat tissues, leading to the reduction in the fat mass, which contributed to the reduction in body weight, serum free fatty acid and leptin.

### Aliskiren improved insulin sensitivity in skeletal muscle

**Figure 6:**
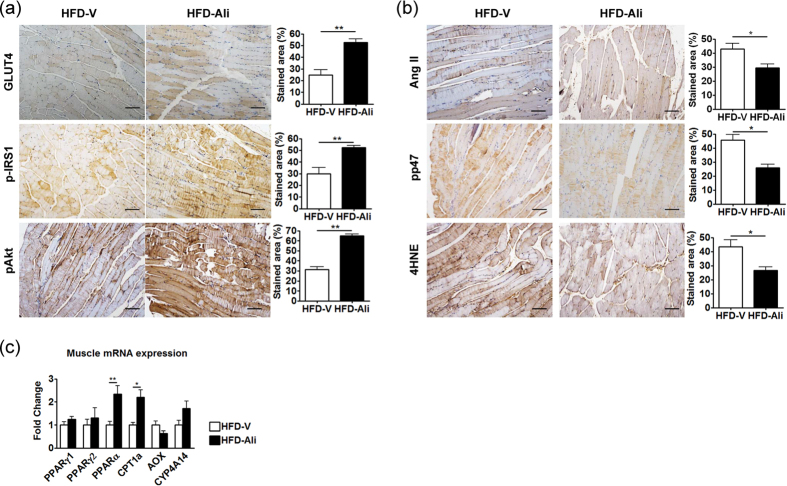
Aliskiren increased insulin sensitivity and upregulated expression levels of PPARα and CPT1a in muscle with reduction in Ang II-associated oxidative stress. (**a**) immunohistochemical images of type4 glucose transporter (GLUT4), phosphor-insulin receptor substrate 1 (p-IRS-1), and phosphor-Akt in the gastrocnemius muscle (scale bar = 100 μm). (**b**) immunohistochemical images of angiotensin II (Ang II), phosphorylated p47 (pp47), and 4-hydroxynonenal (4HNE) (scale bar = 100 μm), and (**c**) RT-PCR of PPARα/γ1/γ2, CPT1a, AOX, and CYP4A14 in the HFD-V and HFD-Ali groups. The results expressed were relative to the HFD-V group, which was arbitrarily assigned a value of 1. *p < 0.05 vs. the HFD-V group; **p < 0.01 vs. the HFD-V group.

Skeletal muscle is the major insulin-sensitizing tissue and an important site for insulin-stimulated glucose utilization. Thus, the expression levels of type 4 glucose transporter (GLUT4), phosphor-insulin receptor substrate 1 (p-IRS-1), and phosphor-Akt were measured. Interestingly, aliskiren treatment significantly increased expression levels of GLUT4, p-IRS1 and p-Akt in the gastrocnemius muscle (Fig. 6a). It indicates that aliskiren may stimulate glucose utilization and increase insulin sensitivity in skeletal muscle, contributing to the improvement of systemic insulin resistance and reduction in serum glucose levels in HFD-fed mice.

Moreover, aliskiren reduced the expression levels of Ang II, pp47 and 4HNE in the gastrocnemius muscle (Fig. 6b). However, the PPARγ level remained unchanged after aliskiren treatment (Fig. 6c). Interestingly, the transcript expression of PPARα and CPT1a increased significantly in the aliskiren-treated gastrocnemius muscle (Fig. 6c). It suggests that the fatty acid oxidation in skeletal muscle may be increased, contributing to the reduction in serum free fatty acids.

## Discussion

This is the first study demonstrating that aliskiren attenuated hepatic steatosis in mice fed with a HFD. Aliskiren down-regulated lipogenic genes in the liver and epididymal fat, activated fatty acid oxidation genes in the liver and skeletal muscle and improved systemic insulin resistance though increasing glucose utilization in skeletal muscle. A few studies have investigated the effect of aliskiren on steatosis in different murine models and the results were inconsistent. Two of them found that aliskiren did not reduce hepatic steatosis in db/db mice^14^ and the C57BL/6 mice fed with a HFD^18^ (the routes and dosages of aliskiren were 25 mg/kg/day via mini-pumps subcutaneously and 50 mg/kg/day orally, respectively). However, in the current and other studies, aliskiren significantly decreased hepatic steatosis in C57BL/6 mice fed with a HFD or MCD^10^ diet and in fatty liver Shionogi-ob/ob mice^9^ (the routes and dosages of aliskiren were 50 mg/kg/day via mini-pumps subcutaneously and 100 mg/kg/day orally, respectively). In fact, the oral bioavailability of aliskiren is low (around 2%)^19^ and transdermal delivery of aliskiren increases its bioavailability by 4- to 54-fold compared to the oral route^20^. Therefore, it is suggested that sufficient bioavailability of aliskiren by an adequate dose or route is necessary to effectively reduce hepatic steatosis.

Kishina *et al*.^9^ reported that aliskiren reduced hepatic steatosis in Shionogi-ob/ob mice, however they only measured the hepatic mRNA expressions of SREBP1c, MTP and PPAR-α, and no differences were found between the aliskiren and control groups. It has been reported that genetic disruption of renin-angiotensin system components^5^,^6^,^7^ reduces hepatic steatosis in rodent models, implying that angiotensin II plays a role in fat accumulation in the liver. In the MCD mice of our previous study, angiotensin II levels in the liver were increased. Aliskiren treatment suppressed hepatic angiotensin II and stimulated fatty acid oxidation-related genes such as PPARα, CYP4A, CPT1a in the liver compared to the vehicle-treated MCD mice^10^. Similarly, in this study, the angiotensin II levels in the liver of the HFD mice were reduced after aliskiren treatment. Interestingly, aliskiren not only enhanced expression levels of fatty acid oxidation-related genes such as CYP4A and CPT1a but also down-regulated lipogenic gene, PPARγ. In fact, the underlying mechanisms of hepatic steatosis in these animal models are different; it seems that aliskerin may act on a particular pathway depending on the cause of hepatic steatosis development.

In this study, aliskiren decreased PPARγ1 and PPARγ2 in the liver of HFD-Ali mice. Both PPAR γ1 and PPARγ2 are expressed at low levels in normal liver, and exposure to the high fat diet in mice has been reported to up-regulate mainly PPARγ2 mRNA in steatotic liver^21^. Similarly, the HFD-V liver had a nearly 18-fold increase in PPARγ2 mRNA compared to the N-V liver in the current study. PPARγ2 is able to induce lipid accumulation in hepatocytes by selectively inducing several adipogenic and lipogenic genes such as ADRP, CD36, FAS, and ACC^22^,^23^,^24^, leading to increased hepatic de novo lipogenesis and hepatic fat accumulation. In the current study, the HFD-V liver exhibited higher expressions of ADRP, CD36, FAS and ACC. After aliskiren treatment, the expressions of ADRP, CD36, FAS and ACC were lowered with a concomitant reduction in PPARγ2 in the HFD-Ali group. Therefore, aliskiren reduced hepatic steatosis by inactivating a PPARγ2-mediated pathway.

Several upstream signaling molecules may mediate the expression of PPARγ. Activation of Akt and ERK has been reported to enhance PPARγ expression^25^,^26^ whereas CREB and AMPK have been reported to inhibit hepatic PPARγ expression to suppress lipid storage and synthesis^27^,^28^. In this study, the HFD-Ali liver had decreased phosphorylation levels of Akt and AMPK. The reduction of AMPK may be attributed to the decreased circulating leptin^29^ and the decreased circulatory insulin could contribute to the decreased phosphorylation of Akt in the liver of the HFD-Ali mice^30^. Briefly, the downregulation of PPARγ in liver may be the consequence of reduction in the insulin/Akt pathway, not the leptin/AMPK pathway.

Interestingly, aliskiren treatment reduced body weight, epididymal fat mass and serum leptin levels in this study without affecting diet intake of the HFD mice, which is similar to previous studies^5^,^31^. The reduction in circulating leptin level was probably due to the decreased adipose fat mass. In addition, because Ang II is involved in adipocyte differentiation, Stucchi *et al*.^31^ suggested that the reduction in the local expression of tissue angiotensin II in peripheral adipose tissues after aliskiren treatment might contribute to the reduction of local fat weight and mass. Similarly, in the current study, the Ang II level was reduced in epididymal fat. We further investigated the expressions of genes involving lipid metabolism. In contrast to the findings in the liver, aliskiren had no effect on fatty acid oxidation-related genes, but down-regulated several lipogenic genes such as SREBP1c, ChREBP and PPARγ2. The decreased expressions of lipogenic genes may be attributed to the decreased circulatory insulin. Furthermore, PPARγ promotes adipocyte differentiation and adipocyte hypertrophy which determine fat mass^32^,^33^ and increased fat mass with large adipocytes contributes to insulin resistance^34^. In addition, long-term activity of PPARγ in adipose tissue may lead to insulin resistance when being fed with a high caloric diet^33^,^35^. Kubota *et al*. reported that heterozygous PPARγ-deficient mice have lower insulin resistance, smaller fat mass and adipocyte size under a HFD compared to PPARγ^+/+^ mice^33^. Therefore, inactivation of insulin/PPARγ pathway in epididymal fat tissue by aliskiren may reduce fat mass and adipocyte hypertrophy, thereby contributing to a decrease in body weight and insulin resistance.

In this study, the action of aliskiren on the skeletal muscle played a critical role to improve systemic insulin resistance. Lastra *et al*. first showed that aliskrein-induced reduction in oxidative stress may improve insulin signaling in muscle^13^. Csibi *et al*. depicted that Ang II inhibited GLUT4 translocation and Akt activation in the skeletal muscle^36^. Thus, in this study, aliskiren-induced Ang II reduction may reverse the GLUT4 expression and attenuate oxidative stress to improve insulin resistance in muscle^37^, which manifested with increased phosphorylation of IRS-1 and Akt in the HFD-Ali group. These effects leaded to amelioration of systemic insulin resistance, subsequently reducing fat accumulation in liver and peripheral fat tissues.

There are limitations in this study. First, it has been reported that the renin knockout mice are lean, insulin sensitive, and resistant to diet-induced obesity without changes in food intake due to a higher metabolic rate and gastrointestinal loss of dietary fat^5^. However, we did not measure energy expenditure and fecal fat content because of lack of equipment. Second, why aliskiren had different mechanistic profiles in liver, fat and muscle remained unclear. Different expressive levels of Ang II in different tissues may lead to the discrepancy; however, further studies are needed to elucidate the underlying mechanisms.

In conclusion, aliskiren attenuated hepatic steatosis in mice fed with a HFD. Aliskiren increased insulin sensitivity and glucose transport activity in skeletal muscle of the HFD mice, contributing to amelioration of systemic insulin resistance. The reduced circulatory insulin inhibited expressions of lipogenic genes in the liver and epididymal fat, leading to decrease in hepatic steatosis and epididymal fat mass. Furthermore, aliskiren activated the fatty acid oxidation genes in the liver and skeletal muscle, which might play a role in lowering serum fatty acids and steatosis. Therefore, the clinical use of aliskiren treatment in patients with NAFLD may be promising.

## Materials and Methods

### Animals

Adult male C57BL/6 mice aged 6–8 weeks purchased from (BioLasco Taiwan Co., Ltd., Taipei, Taiwan) were used in all experiments. All of the mice were caged at 22 °C with a 12-hour light-dark cycle, and allowed free access to food (Laboratory Autoclavable Rodent Diet 5010) or a high-fat diet (HFD) (D12492; containing 34.9% fat (mostly saturated) by weight, which yields ∼60% calories from fat and has a caloric density of 5.24 kcal/g) and water. This study was approved by the Animal Experiment Committee of Taipei Veterans General Hospital and performed according to the “guides for the care and use of laboratory animals” prepared by the National Academy of Science, USA.

### Protocol

The mice were divided into four groups. Hepatic steatosis, obesity and insulin resistance were induced by feeding with a HFD (n = 14). After 4 weeks of feeding, the HFD mice or mice fed with a normal chow diet were randomly divided to receive aliskiren (50 mg/kg/day for 4 weeks) via ALZET osmotic minipumps (1004 type, DURECT Corporation, Cupertino, CA) (N-Ali/HFD-Ali groups, n = 6/7) or vehicle (N-V/HFD-V groups, n = 6/7). Under zoletil anesthesia, minipumps filled with aliskiren (Novartis, Basel, Switzerland) or vehicle (double distilled water, 100 μL) were implanted subcutaneously into the right upper back of the mice. The dose of aliskiren we chose was according to a previous study^38^ which showed sufficient inhibition of renin activity in mice *in vivo*. In addition, we previously found that this dosage can decrease chronic liver injury in MCD mice^10^.

After feeding with a HFD for 8 weeks, all groups of mice were sacrificed. After an overnight fasting, blood samples were collected by submandibular venipuncture. Serum was separated by refrigerated centrifugation and stored at −80 °C until assay. The liver, epididymal fat, and gastrocnemius muscle were rapidly excised after phosphate-buffered saline perfusion, rinsed in ice-cold saline, and then weighed. Aliquots of tissues were snap frozen in liquid nitrogen and kept at −80 °C until being analyzed. A portion of each tissue was fixed in 10% formalin for histology.

### Biochemistry measurements

Levels of serum glucose, aspartate aminotransferase, alanine aminotransferase, and triglyceride were measured using a standard auto SMAC analyzer (Roche Diagnostics GmbH, Mannheim, Germany). Serum free fatty acid was measured using a Free Fatty Acid Assay Kit (Zen-Bio, Inc., Durham, NC).

### Measurement of serum insulin, leptin, and adiponectin

Serum insulin was measured using an Ultra-Sensitive Mouse Insulin ELISA Kit (Crystal Chem Inc., Downers Grove, IL), and serum leptin and adiponectin levels were measured using ELISA kits (Abcam, Cambridge, UK).

### Glucose and insulin tolerance tests

Another set of HFD-V/HFD-Ali mice (n = 4/4) received glucose and insulin tolerance tests. After a 16-hour fast, glucose tolerance tests were performed by intraperitoneal injection of D-glucose (Sigma-Aldrich, Inc. St. Louis, MO) at a dose of 2.0 mg/g body weight. For insulin tolerance tests, mice were injected with regular human insulin (Santa Cruz Biotechnology, Inc., CA) at a dose of 0.75 U/kg body weight after a 6-hour fast. Blood glucose was measured using a portable blood glucose meter (one touch ultra2, life scan, Johnson&Johnson, USA).

### Measurement of hepatic steatosis

Liver steatosis was measured by Oil Red O staining on frozen tissues (8 μm). Hepatic triglyceride levels were assessed using a commercially available kit (Cayman Chemical Company, Ann Arbor, Michigan).

### Real-time quantitative reverse transcriptase-polymerase chain reaction (PCR)

The nucleotide sequences of the primers used in this study are shown in Table 1. Quantitative gene expressions were measured on an ABI PRISM 7900HT Sequence detection system (Applied Biosystems Inc. Foster City, CA) using SYBR Green. The specificity of each PCR product was evaluated by melting curve analysis, followed by agarose gel electrophoresis.

### Western blot analysis

Protein extraction was performed according to standard methods, and nuclear protein was extracted using NE-PER Nuclear extraction reagents (Thermo Scientific, Waltham, MA). The blots were incubated with the primary antibodies shown in Table 2. After washing, the membranes were incubated with horseradish peroxidase-conjugated goat anti rabbit (Jackson ImmunoResearch Laboratories Inc., West Grove, PA) for the rabbit primary antibodies for 1 hour. Subsequently, the blots were developed by enhanced chemiluminescence (ECL Western Blotting Analysis System, Amersham, UK). The intensities of the bands of interest were analyzed using Image J software (National Institutes of Health, Bethesda, MD).

### Histological studies

The tissues were fixed for 24 hours with 10% paraformaldehyde at room temperature, then dehydrated, embedded in paraffin, cut into 4-μm-thick slices, and stained with hematoxylin and eosin (H&E).

For immunohistochemical staining, the tissues were sliced into 4-μm-thick sections and transferred to a 10 mmol/L citrate buffer solution at pH 6.0 or proteinase K to retrieve antigens. After washing in phosphate-buffered saline, 3% H_2_O_2_ was applied to the slides for 10 minutes to block endogenous peroxidase activity. The slides were then incubated at room temperature with 5% heated bovine serum albumin for 30 minutes, followed by incubation at 4 °C overnight with the primary antibodies shown in Table 2. After overnight incubation, the slides were incubated with a secondary antibody for 30 minutes, and then colored using a super-sensitive polymer-HRP IHC detection system (BioGenex Laboratories Inc., Fremont, CA), followed by counterstaining with Mayer’s hematoxylin. Negative control sections were stained under identical conditions except that a buffer solution was substituted for the primary antibody.

### Statistical analysis

Data were analyzed using GraphPad Prism 4 (GraphPad Software, San Diego, CA) and expressed as means ± S.E.M. Statistical significance in each group was determined using one-way ANOVA with post hoc multiple comparisons performed using the Newman-Keuls test. When criteria for parametric testing were violated, the Mann-Whitney U-test was performed. Protein levels between the two paired groups were compared using the Student’s t-test. Significance was determined at a p-value less than 0.05.

## Additional Information

**How to cite this article**: Lee, K.-C. *et al*. Aliskiren Reduces Hepatic steatosis and Epididymal Fat Mass and Increases Skeletal Muscle Insulin Sensitivity in High-Fat Diet-Fed Mice. *Sci. Rep.* **6**, 18899; doi: 10.1038/srep18899 (2016).

## Figures and Tables

**Table 1:** Primer pairs used for quantitative real time PCR.

**Gene name**	**Primer**	**Sequence**	**Size**
FATP1	forward	5′-CGC TTT CTG CGT ATC GTC TG -3′	120
	reverse	5′-GAT GCA CGG GAT CGT GTC T -3′	
FATP4	forward	5′-GAT GGC CTC AGC TAT CTG TGA -3′	202
	reverse	5′-GGT GCC CGA TGT GTA GAT GTA -3′	
ChREBP	forward	5′-GGA CAA GAT CCG GCT GAA CA -3′	131
	reverse	5′-CAG GTT TCC GGT GCT CAT CT -3′	
LXRα	forward	5′- ATC GCC TTG CTG AAG ACC TCT G -3′	159
	reverse	5′- GAT GGG GTT GAT GAA CTC CAC C -3′	
SREBP1c	forward	5′- CTG GGG GTG AGA CAG GGG AC -3′	145
	reverse	5′- GAT GGT GGA GGG GAC AAG GG -3′	
PPAR-γ1	forward	5′- GGACTGTGTGACAGACAAGATTTG -3′	52
	reverse	5′- CTGAATATCAGTGGTTCACCGC -3′	
PPAR-γ2	forward	5′-TGG GTG AAA CTC TGG GAG AT -3′	454
	reverse	5′-CCA TAG TGG AAG CCT GAT GC -3′	
CYP4A14	forward	5′-CCCTGCTCCGCTTTGAATTG -3′	135
	reverse	5′-AGCTGCCCTGACTCCATCA -3′	
CPT1a	forward	5′-CGC ACG GAA GGA AAA TGG -3′	211
	reverse	5′-TGT GCC CAA TAT TCC TGG -3′	
AOX	forward	5′- CTT GTT CGC GCA AGT GAG G -3′	213
	reverse	5′- CAG GAT CCG ACT GTT TAC C-3′	
PPARα	forward	5′- CCG AAC ATT GGT GTT CGC AG -3′	161
	reverse	5′- AGA TAC GCC CAA ATG CAC CA -3′	
MTTP	forward reverse	5′- CGT CCA CAT ACA GCC TTG AC -3′ 5′- CCA CCT GAC TAC CAT GAA GC -3′	111
CD36	forward reverse	5′- CTTCCACATTTCCTACATGCAA -3′ 5′- ATCCAGTTATGGGTTCCACATC -3′	109
ADRP	forward reverse	5′- CTT GTG TCC TCC GCT TAT GTC AGT-3′ 5′- CTG CTC CTT TGG TCT TAT CCA CCA-3′	350
GAPDH	forward	5′- TGT TGA AGT CGC AGG AGA CAA CCT-3′	111
	reverse	5′- AAC CTG CCA AGT ATG ATG ACA TCA -3′	

**Table 2:** Antibody details and conditions used for Western blotting and immunohistochemistry.

**Antibody**	**Supplier**	**Catalog no.**	**Application**	**Dilution**
β-actin	Novus	NB600-501	WB	1:5000
Lamin B1	Santa cruz	sc-6216	WB	1:1000
PPARγ	Cell Signaling	#2435	WB/IHC	1:1000/1:400
t-ERK	Cell Signaling	#9102	WB	1:1000
p-ERK	Cell Signaling	#9101	WB	1:1000
t-AMPK	Cell Signaling	#2532	WB	1:1000
p-AMPK	Cell Signaling	#2535	WB	1:1000
t-Akt	Cell Signaling	#9272	WB	1:1000
p-Akt	Cell Signaling	#4058	WB	1:1000
t-CREB	Cell Signaling	#9197	WB	1:1000
p-CREB	Cell Signaling	#9198	WB	1:1000
FAS	Cell Signaling	#3180	IHC	1:100
ACC	Cell Signaling	#3676	IHC	1:200
Ang II	phoenix pharmaceuticals	H-002-12	IHC	1:2000
p-p47	Assay biotech	A1171	IHC	1:2500
4HNE	Alpha diagnostic	HNE11-S	IHC	1:2500
GLUT4	Santa cruz	sc-7938	IHC	1:200
p-IRS1	Santa cruz	sc-17200	IHC	1:200
p-Akt	Epitomics	#2118-S	IHC	1:100

Antibodies were purchased from Cell signaling (Beverley, MA), Novus biological (Littleton, CO), Santa cruz (Santa Cruz Biotechnology, Inc., CA), phoenix pharmaceuticals (Burlingame, CA), Assay biotech (Sunnyvale, CA), Alpha diagnostic (San Antonio, TX), Epitomics (Burlingame, CA). WB: western blotting; IHC: immunohistochemistry.
